# Facile Preparation of Metal-Organic Framework (MIL-125)/Chitosan Beads for Adsorption of Pb(II) from Aqueous Solutions

**DOI:** 10.3390/molecules23071524

**Published:** 2018-06-25

**Authors:** Xue-Xue Liang, Nan Wang, You-Le Qu, Li-Ye Yang, Yang-Guang Wang, Xiao-Kun Ouyang

**Affiliations:** School of Food and Pharmacy, Zhejiang Ocean University, Zhoushan 316022, China; 13665804509@163.com (X.-X.L.); ynwangnan@163.com (N.W.); liyey@zjou.edu.cn (L.-Y.Y.); ygw0510@sohu.com (Y.-G.W.)

**Keywords:** metal-organic framework, chitosan beads, adsorption, Pb(II)

## Abstract

In this study, novel composite titanium-based metal-organic framework (MOF) beads were synthesized from titanium based metal organic framework MIL-125 and chitosan (CS) and used to remove Pb(II) from wastewater. The MIL-125-CS beads were prepared by combining the titanium-based MIL-125 MOF and chitosan using a template-free solvothermal approach under ambient conditions. The surface and elemental properties of these beads were analyzed using scanning electron microscopy, Fourier transform infrared and X-ray photoelectron spectroscopies, as well as thermal gravimetric analysis. Moreover, a series of experiments designed to determine the influences of factors such as initial Pb(II) concentration, pH, reaction time and adsorption temperature was conducted. Notably, it was found that the adsorption of Pb(II) onto the MIL-125-CS beads reached equilibrium in 180 min to a level of 407.50 mg/g at ambient temperature. In addition, kinetic and equilibrium experiments provided data that were fit to the Langmuir isotherm model and pseudo-second-order kinetics. Furthermore, reusability tests showed that MIL-125-CS retained 85% of its Pb(II)-removal capacity after five reuse cycles. All in all, we believe that the developed MIL-125-CS beads are a promising adsorbent material for the remediation of environmental water polluted by heavy metal ions.

## 1. Introduction

With the development of modern industry, the standards of living have been continuously improving. However, this has also led to many environmental problems, including the heavy metal pollution of water, which creates risks for human health, the environment and ecological systems [1]. Most specifically, lead can enter the body through contaminated food and the respiratory tract in the forms of vapor, dust and chemicals [2]. Moreover, the amount of lead absorbed by the body from food and water increases with age [3]. Lead poisoning mainly damages the nervous and hematopoietic systems, as well as the kidneys, but can also affect the functions of the circulatory and reproductive systems, and can even cause cancer. In addition, lead has been reported as teratogenic and mutagenic [4]. Since the lead pollution problem is growing, it is important to develop a highly efficient adsorbent for the remediation of Pb(II) [5]. Many methods, mostly based on physical and chemical processes, for the removal of Pb(II) from polluted environmental water have been described in the literature [6]. Among these, the adsorption of Pb(II) has proven to be the best treatment approach owing to several significant advantages that include design simplicity, cost efficiency, ease of operation and absence of secondary pollution [7].

With the rapid development of new materials, metal-organic frameworks (MOFs) have received an increasing amount of attention in recent years [8]. MOFs are crystalline porous materials that have periodic network structures formed by the self-assembly of transition metal ions and organic ligands [9]. They not only have many excellent features such as high porosity, low density, large surface area and adjustable aperture, but are also topologically diverse and scalable [10]. In addition, MOFs provide significant advantages to the fields of gas storage, small-molecule separation and catalysis due to their special structural properties and ability to change their internal structures [11]. Indeed, MOFs have been shown to favorably adsorb species that include heavy metals and drugs.

The development of titanium-based MIL-125 represents an important breakthrough in the use of metal nodes as functional moieties [12]. Various modifications of MIL-125 have been reported in recent years. One example is the highly crystalline NH_2_-MIL-125, which exhibits good sorption-isotherm-model behavior and high water capacity for an adsorbent-heat-transformation system [13]. Although titanium based metal organic framework MIL-125 can remove Pb(II) well and separate it from waste-water simply by centrifugation, this method has proven to be troublesome, which has restricted its application [14].

Chitosan (CS) is a natural and completely biodegradable polymeric material that is very attractive to researchers because of its versatile chemical and physical properties. Chitin is the raw material for chitosan and is produced from chitinous solid waste from the food industry [15]. Chitosan is obtained by chitin deacylation and has broader application prospects than chitin since it contains a larger number of chelating amino groups that can be modified [16]. Even though this valuable structure contains reactive hydroxyl and amino groups that can potentially bind heavy metals, as well as various polymers, chitosan derivatives have proven to be more effective than the pure form [17,18]. Reportedly, chitosan can easily be processed into membranes, nanofibers, beads, microparticles and nanoparticles. Moreover, the outstanding biological properties of this molecule have led to its enormous importance in a variety of pharmaceutical and biomedical applications [19]. However, despite all of its attractive features, in its pure form, chitosan adsorbs Pb(II) poorly [20].

In this study, we synthesized MIL-125 using the hydrothermal-solvent method and mixed it with chitosan to form solidified beads in a sodium tripolyphosphate solution. The synthesized MIL-125-CS beads contained carboxyl and hydroxyl groups derived from chitosan, and its bead structure facilitated the separation from water in subsequent experiments. Furthermore, chitosan wraps on the surface of MIL-125 were found to enhance the stability of the beads in water. Finally, the abilities of the MIL-125-CS beads to adsorb Pb(II) from polluted water were tested. Analysis of the Pb(II) levels revealed that the beads exhibited good adsorption capacities and solid-liquid separation characteristics. In addition, filtration or centrifugation was not required during the recovery of the MIL-125-CS beads.

## 2. Results and Discussion

### 2.1. Synthesis and Characterization of MIL-125-CS

**Figure 1:**
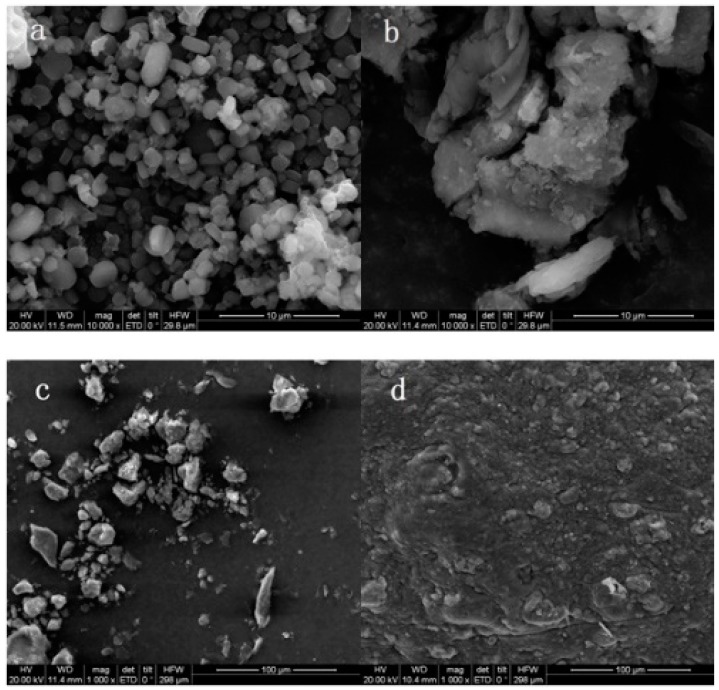
SEM pictures of (**a**) MIL-125(Ti), (**b**,**c**) MIL-125-CS and (**d**) MIL-125-CS-Pb.

**Figure 2:**
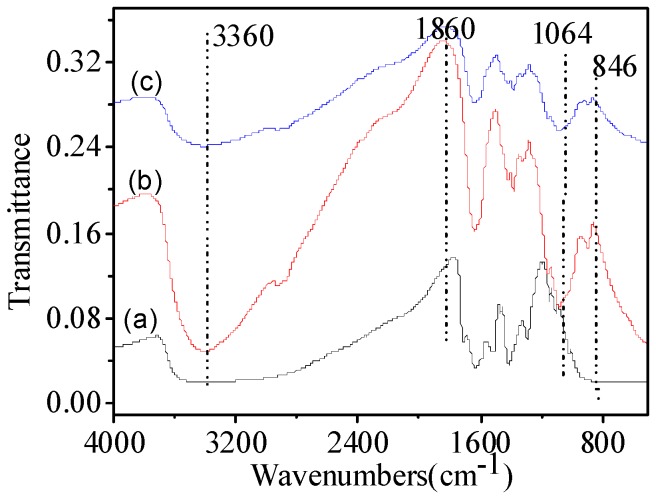
FTIR spectra of (**a**) MIL-125, (**b**) MIL-125-CS and (**c**) MIL-125-CS-Pb.

SEM pictures of MIL-125, the MIL-125-CS beads and Pb(II) loaded on MIL-125-CS (MIL-125-CS-Pb) are displayed in Figure 1. The SEM image in Figure 1a revealed that MIL-125 had an octahedral structure with a size range of 5–20 um and smooth surfaces [21], thereby verifying that MIL-125 had been successfully synthesized. A comparison of the SEM pictures of pure MIL-125 (Figure 1a) and the MIL-125-CS beads (Figure 1b,c) revealed that the surface of MIL-125 had undergone some changes. Notably, the surface of the MIL-125-CS beads was rougher and denser than that of MIL-125, which was attributed to the assembly of chitosan on the MIL-125 layers. Figure 2c displays a half-cut view of a dry MIL-125-CS bead that shows Pb(II) successfully loaded on the MIL-125-CS surface. This result clearly indicates that the surface of MIL-125-CS becomes rougher after the Pb(II) loading.

FTIR spectra of MIL-125, MIL-125-CS and MIL-125-CS-Pb are shown in Figure 2. The band observed in Figure 2a at 3360 cm^−1^ corresponded to the O–H stretching vibrations, while the band at 1860 cm^−1^ was ascribed to the carboxyl C=O moiety. Similarly, the bands at 3360 cm^−1^ in both spectra shown in Figure 2b were attributed to the O–H vibrations. In addition, the adsorption peak at 2900 cm^−1^ corresponded to the C–H stretching vibrations based on MIL-125-CS, while those at 1162 and 1064 cm^−1^ were assigned to the C–C stretching vibrations, proving that MIL-125-CS contained MIL-125. In addition, the band 1210 cm^−1^ was ascribed to the O-Ti-O vibration [22] . Furthermore, as can be seen from Figure 2a,b, the key peaks in the spectrum of MIL-125 can also be detected in that of MIL-125-CS, and the band between 1000 and 846 was ascribed to -NH of chitosan, which indicates that MIL-125-CS was successfully synthesized. Furthermore, the peak shift at 1665–1600 cm^−1^ (-NH_2_ bending mode) reflected the interaction between the Fe^3+^ ion and -NH_2_ group [23]. Moreover, the hydroxyl and carboxyl bands in the spectrum of MIL-125-CS-Pb were better defined than those in the spectrum of pure MIL-125-CS, which implied that Pb(II) was embedded through interactions with both the O–H and COO− units [24]. Reportedly, the stretching and torsional vibrations of these functional groups weaken with the increasing ionic volume, thus resulting in an altered adsorption maximal [25]. Through the aforementioned analysis, we concluded that Pb(II) reacted chemically with MIL-125-CS and caused changes in the infrared adsorption peaks, thereby demonstrating that Pb(II) was successfully adsorbed onto the surfaces of the MIL-125-CS beads.

**Figure 3:**
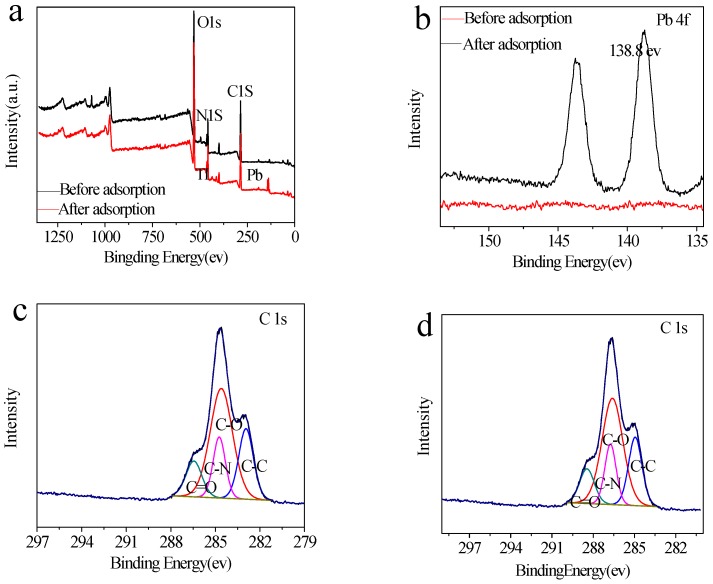
XPS survey spectra of (**a**) MIL-125/CS and MIL-125/CS-Pb, (**b**) Pb 4f, (**c**) C1s of MIL-125/CS and (**d**) C1s of MIL-125/CS-Pb.

XPS was used to investigate the chemical compositions of MIL-125-CS and MIL-125-CS-Pb. In particular, Ti, C, O, N and Pb were found to be present in the adsorbent exposed to Pb(II). The XPS survey, Pb 4f and C 1s spectra of MIL-125-CS and Pb(II)-loaded MIL-125-CS are shown Figure 3. The Pb(II) adsorption peak that appears in the survey spectrum of MIL-125-CS-Pb (Figure 3a) confirms that Pb(II) was indeed present on MIL-125-CS. Moreover, the Pb 4f spectrum exhibits a binding energy of 138.8 eV (Figure 3b) assigned to Pb 4f_7/2_, further verifying that Pb(II) was loaded on MIL-125-CS and suggesting that lead carbonate and lead oxide were possibly formed during the adsorption process [26]. In contrast, binding energies of 284.8, 286.581, 285.521 and 288.185 eV were observed in the C 1s spectrum of MIL-125-CS shown in Figure 3c, which were ascribed to the C–C, C–O, C–N and C=O groups, respectively, of MIL-125-CS. Meanwhile, Figure 3d revealed important differences in the signals corresponding to the main functional groups such as C–O and C=O, with the peaks corresponding to these groups shifted to 286.468 and 288.254 eV, respectively. All in all, the XPS analysis clearly indicates that carboxylate groups played a significant role in the Pb(II) adsorption process.

**Figure 4:**
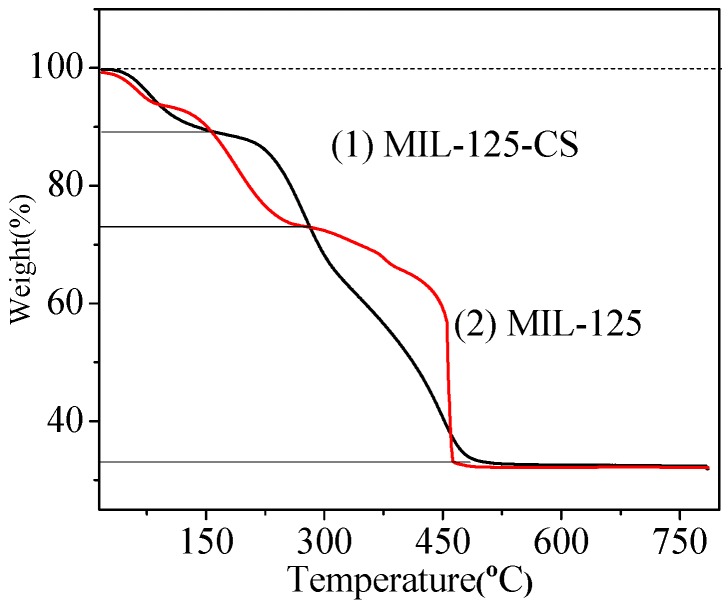
TGA curves of (1) MIL-125-CS and (2) MIL-125.

TGA examines the stabilities and compositions of the materials being studied, in this case MIL-125 or MIL-125-CS, through programmed temperature changes. Figure 4 displays TGA traces of MIL-125-CS and MIL-125, which revealed that temperature increases led to weight losses. The first weight-loss step was associated with the vaporization of water from the sample at ambient temperature (0–180 °C). The weight losses of MIL-125-CS and MIL-125 were about 11%, which was due to the decomposition of guest elements adsorbed on the adsorbent. The further degradation step was associated with a series of processes. Overall, the weight of the MIL-125-CS sample declined faster than that of MIL-125, which was ascribable to the large number of hydroxyl functional groups in chitosan that are easily dehydrated with the loss of water at higher temperatures. However, by the time both samples had reached 500 °C, they had lost the same amount of weight. Moreover, no further weight losses were observed with the increasing temperature, since chitosan had formed carbide [27], and MIL-125 had transformed into an ultrafine TiO_2_ powder that was not easy to crack at higher temperatures (>500 °C) [28]. The main weight losses observed between 300 and 500 °C were attributed to the degradation of each MOF through the decomposition of the aminoterephthalic acid units in MIL-125, ultimately producing an amorphous TiO_2_ residue [29].

**Figure 5:**
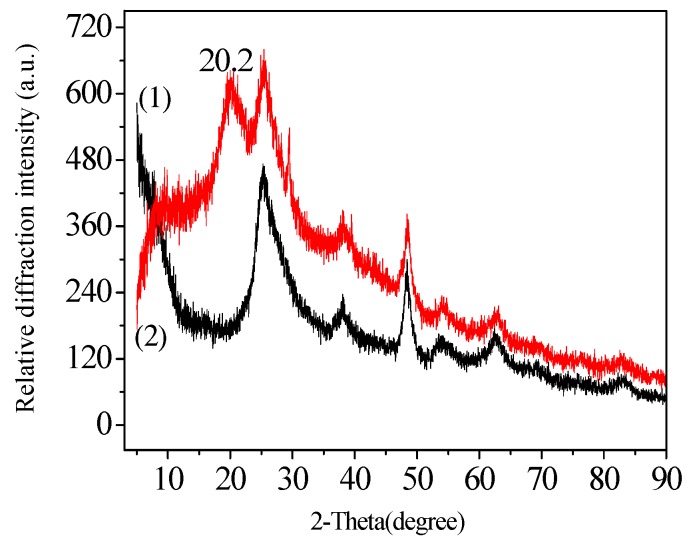
XRD curves of (1) MIL-125 and (2) MIL-125-CS.

The XRD patterns of pure MIL-125 and MIL-125-CS are shown in Figure 5. The diffraction peaks of MIL-125 are consistent with those reported in previous studies [30], which indicated that MIL-125 was prepared successfully. In addition, the XRD pattern of MIL-125-CS was almost the same as that of MIL-125, except for a few changes in the (20.2) diffraction, which may correspond to –NH_2_, and this similarity was due to the fact that CS and MIL-125 have polymerized and that the structure of MIL-125 had not changed.

### 2.2. Adsorption Studies

#### 2.2.1. Effect of the Contact Time

**Figure 6:**
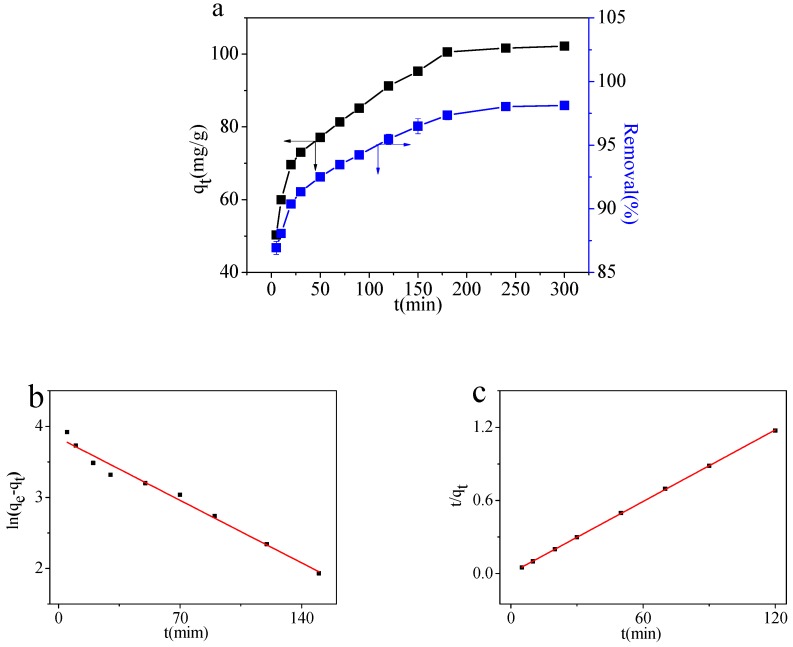
(**a**) Effect of contact time on Pb(II) adsorption capacity of MIL-125-CS. Data fitted to (**b**) pseudo-first-order and (**c**) pseudo-second-order kinetic models.

Contact time experiments were conducted in the time range of 5–300 min during the Pb(II) removal using the MIL-125-CS beads, and the respective adsorption capacities were determined for different adsorption times. As can be observed in Figure 6a, *q_t_* increases rapidly with the increasing adsorption time during the initial stages of adsorption, which was ascribable to unoccupied MIL-125-CS. However, the adsorption of Pb(II) into the MIL-125-CS beads gradually reached equilibrium within 180 min, as evidenced by the fact that no obvious adsorption-capacity changes were noticed after 180 min. Consequently, a contact time of 180 min was applied to the following experiments.

Additionally, we studied the kinetics of the adsorption of Pb(II) by the MIL-125-CS beads. Experiments were conducted in which MIL-125-CS beads (0.5 g) were added to 20-mL aliquots of Pb(II) solutions with different initial concentrations (10–1000 mg/g). During the analysis of the adsorption data, we assumed either a pseudo-first-order or pseudo-second-order kinetics model, the specific rate equations of which are:

(1) $$\ln\left(q_{e}-q_{t}\right)=\ln q_{e}-k_{1}t$$

(2) $$\frac{t}{q_{t}}=\frac{1}{k_{2}q_{e}{}^{2}}+\frac{t}{q_{e}}$$

In these equations, *k*_1_ (min^−1^) and *k*_2_ (g mg^−1^ min^−1^) are the adsorption rate constants from the two kinetics models, respectively. Figure 6b,c shows the experimental data fitted to these models, with the resulting parameters provided in Table 1.

The rate constant data for the adsorption process fit the pseudo-second-order kinetic model (*R*^2^ = 0.9999) better than the pseudo-first-order model (*R*^2^ *=* 0.97978). In addition, the pseudo-second-order-calculated *q_e_* value of 102.04 mg/g is in good agreement with the experimentally-determined value of 99.94 mg/g. Hence, we concluded that the Pb(II) adsorption onto the MIL-125-CS beads follows the pseudo-second-order kinetics, which is consistent with the chemical-adsorption process [31].

#### 2.2.2. Effect of pH

**Figure 7:**
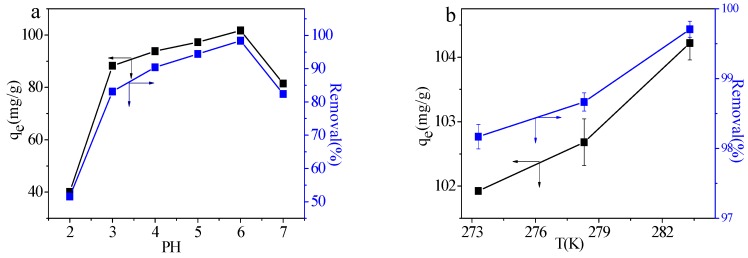
The effect of the Pb(II)-solution pH (**a**) and reaction temperature (**b**) on Pb(II) adsorption on MIL-125-CS beads.

Additionally, we investigated the effect of the initial Pb(II)-solution pH on the adsorption by the MIL-125-CS beads and summarize the results in Figure 7a. As can be seen, the pH greatly influenced the adsorption properties of the beads toward Pb(II). The *q_e_* for the Pb(II) adsorption was observed to increase from 40.1 ± 0.57–101.75 ± 0.67 mg/g, as the pH increased from 2–6, respectively. Therefore, it is clearly more beneficial to remove Pb(II) with MIL-125-CS at a higher pH. This phenomenon was attributed to the prolific H^+^ ions in the solution that compete with Pb(II) for loading onto the MIL-125-CS beads [32]. At the same time, the functional groups on the surfaces of the MIL-125-CS beads were protonated, thus leading to a positively-charged adsorbent surface that resulted in a decreased tendency to adsorb activated Pb(II). However, the adsorption capacity clearly dropped sharply as the pH was increased from 6–7. This observation was attributed to the hydroxide (OH^−^) and ferric (Fe^2+^) ions reacting to form a precipitate. Finally, we conclude that at low values, the pH had a significant impact on the Pb(II)-adsorption process, with a maximum *q_e_* observed at pH 6. As a result, subsequent tests were carried out at pH 6, at which hydrolysis and Pb(II) sediment formation can be avoided.

#### 2.2.3. Effect of the Temperature

The effect of the temperature on the Pb(II)-adsorption capacity of the MIL-125-CS beads is illustrated in Figure 7b. In this experiment, MIL-125-CS (0.5 g) was mixed into 20 mL of a 200-mg/g Pb(II) solution, after which it was shaken at the required temperature until reaching an adsorption equilibrium. The relationship between the adsorption capacity and temperature was clearly evident, with the adsorption capacity increasing from 101.9–104.1 mg/g with the increasing temperature. Therefore, we concluded that the process of loading Pb(II) onto the MIL-125-CS beads was endothermic [33].

In order to provide insight into the mechanism of the adsorption process, the changes in *C_e_*/*q_e_* were used to determine the changes in Gibbs free energy (Δ*G*, kJ/mol), enthalpy (Δ*H*, kJ/mol) and entropy (Δ*S*, J/mol.K).These quantities are related by the following formula:

(3) $$\Delta G=-RT\ln\frac{q_{e}}{C_{e}}=-RT\left(-\frac{\Delta H}{RT}+\frac{\Delta S}{R}\right)$$

In this equation, *T* (K) indicates the adsorption temperature and represents the common gas constant. Moreover, Δ*G* was used to establish if the adsorption process is spontaneous and if the thermodynamic temperature is conducive to the Pb(II) loading on the MIL-125-CS beads. Notably, Δ*H* was affected by temperature, with the positive values confirming the spontaneous nature of the experimental process [34]. These values are listed in Table 2.

#### 2.2.4. Effect of Pb(II) Concentration

**Figure 8:**
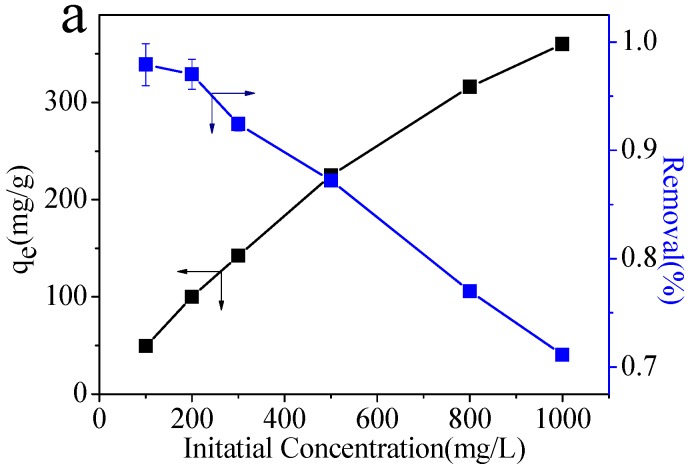

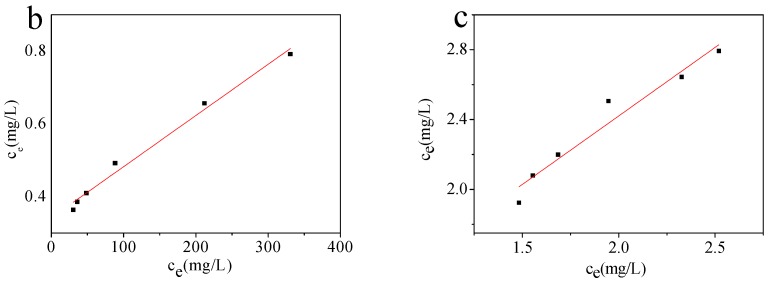
(**a**) The impact of initial Pb(II)-solution concentration (*C*_0_) on the adsorption capacity of the MIL-125-CS beads. Linear fits to the (**b**) Langmuir and (**c**) Freundlich isotherm models.

Batch experiments were carried out in order to determine the impact of the Pb(II) concentration on the adsorption capacity of the MIL-125-CS beads. As illustrated in Figure 8, the adsorption capacity of the MIL-125-CS beads increased from 49.18 ± 0.30% (100 mg/g) to 360.05 ± 3.36% (1000 mg/g) as the initial Pb(II) concentration was increased. While the heavy metal ions were still easily captured by the adsorption sites at higher Pb(II) concentrations, the increase in adsorption capacity gradually decreased, which was attributable to the relatively fewer adsorption sites with the larger *C*_0_ values.

Equilibrium adsorption isotherms are an efficient means of confirming the mechanism associated with the adsorption process of an adsorbent. In this study, we employed the isotherm model to analyze the adsorption-equilibrium curve for Pb(II) interacting with the MIL-125-CS beads. The Langmuir and Freundlich isotherm models are described by the following formulas [35]:

(4) $$\frac{C_{e}}{q_{e}}=\frac{1}{bq_{m}}+\frac{C_{e}}{q_{m}}$$

(5) $$\mathrm{lg}q_{e}=\mathrm{lg}K_{F}+\frac{1}{n}\mathrm{lg}C_{e}$$

where *q_max_* (mg/g) indicates the maximum removal capacity for Pb(II) loaded onto the MIL-125-CS beads, *b* is the Langmuir constant, *K_F_* (mg/g) is the Freundlich adsorption constant and *n* is the Freundlich adsorption constant that is related to the adsorption intensity. These values are listed in Table 3.

Figure 8b,c shows the degree of coincidence between the experimental curves and the two isotherm models. It can be clearly observed that the Langmuir model better describes how Pb(II) interacts with the MIL-125-CS beads. The Langmuir model supposes that Pb(II) is foremost adsorbed as a monolayer on the surface of the MIL-125-CS bead and that the adsorption energies were uniformly distributed over the adsorbent surface [36].

*R_L_* is a dimensionless separation factor that can be determined from the Langmuir model according to the following formula:

(6) $$R_{L}=\frac{1}{1+bC_{0}}$$

The resulting *R_L_* values (0–1, Table 4) indicate that Pb(II) was effectively adsorbed on the surfaces of the MIL-125-CS beads.

### 2.3. Comparison of the Adsorption Capacities of the Adsorbents

Comparison experiments involving the absorption of Pb(II) on MIL-125, the chitosan beads and the MIL-125-CS beads revealed that MIL-125-CS exhibited a *q_e_* of 100.03 mg·g^−1^, which is higher than that of the chitosan beads (60.97 mg·g^−1^) and MIL-125 (94.72 mg·g^−1^). The results also indicated that MIL-125 and the chitosan beads played the same significant role during the adsorption of Pb(II), especially when combined in the composite MIL-125-CS beads. Consequently, the MIL-125-CS beads were used as the adsorbent in subsequent experiments.

### 2.4. Reusability of MIL-125-CS Beads

**Figure 9:**
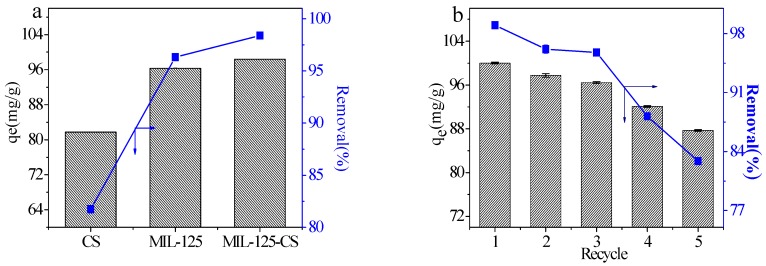
Adsorption capacities and removal ratios (%) of CS, MIL-125 and the MIL-125-CS beads (**a**) and the reusability of the MIL-125-CS beads for Pb(II) adsorption (**b**).

In order to investigate the recycling characteristics of the MIL-125-CS beads, 0.1 mol/L NaOH (desorbent) were added to a mixture of the MIL-125-CS beads in the Pb(II) solution [37]. Following the Pb(II) desorption, Pb(II) was re-adsorbed onto the MIL-125-CS beads. This process was repeated five times (Figure 9b), with the adsorption capacity measured after each cycle. While the adsorption capacity of the MIL-125-CS beads was slightly lower, having dropped from 100.02 ± 0.15%–87.70 ± 0.14% mg/g, after five continuous usage cycles, the Pb(II)-elimination rate was retained at 83.85 ± 0.28%. As a result of the series of experiments and analyses presented in this study, we suggest that the MIL-125-CS beads are prospective materials for efficient Pb(II) removal.

### 2.5. Stability of MIL-125

**Figure 10:**
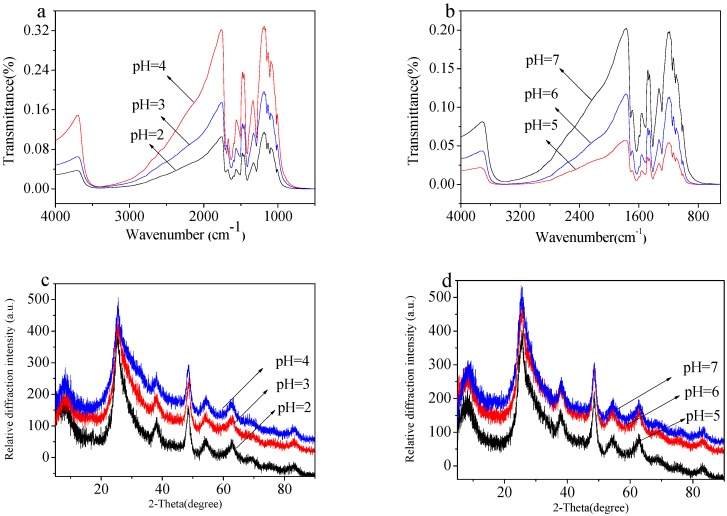
FTIR spectra (**a**,**b**) and XRD patterns (**c**,**d**) of MIL-125 after treatment with solution of different pH.

The structure stability of MIL-125 in a water environment with a pH range of 2–7 was investigated by FTIR and XRD analyses. As can be seen from the FTIR spectra (shown in Figure 10a,b), no obvious difference was observed in MIL-125 after acidity treatment. A few changes were detected in the crystal form of MIL-125 after the acid treatment (shown in Figure 10c,d). Based on these results, we concluded that the crystal structure did not change after the acid treatment, and MIL-125 maintained its stability in a water environment with a pH ranging from 2–7.

## 3. Materials and Methods

### 3.1. Materials

Titanium isopropoxide (C_12_H_28_O_4_Ti, 95%), ferric chloride hexahydrate (FeCl_3_·6H_2_O), benzene-1,4-dicarboxylicacid (BDC), *N*,*N*-dimethylformamide (DMF), sodium tripolyphosphate (Na_5_P_3_O_10_) solution and methanol (CH_3_OH) were provided by Aladdin Reagent Co., Ltd. (Shanghai, China), while chitosan with a viscosity of 100–200 mpa.s and a degree of deacetylation ≥95% was furnished by Aladdin Reagent Co., Ltd. (Shanghai, China). Deionized water was prepared by our laboratory. All reagents were used without further purification.

### 3.2. Preparation of MIL-125 and the MIL-125-CS Beads

MIL-125(Ti) was formed according to a previously-reported method [38]. C_12_H_28_O_4_Ti (14.64 g) and BDC (13.38 mL) were added to a mixture of methanol (24 mL) and DMF (216 mL) and stirred at room temperature until a homogeneous solution was obtained. Then, the mixed solution was placed in a Teflon-lined stainless steel autoclave and heated at 80 °C for 48 h. The reaction was cooled to room temperature and collected by filtration, after which the white precipitate was washed three times with DMF to remove the remaining unreacted titanium isopropoxide from the porous framework. The resulting solid was washed several times with methanol and dried at 80 °C under vacuum for 5 h, and the prepared white solid powder was triturated for subsequent use.

**Scheme 1:**
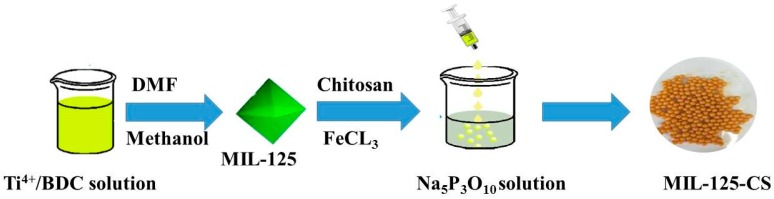
Synthesis of the MIL-125-CS beads. BDC, benzene-1,4-dicarboxylicacid.

The synthesis of the MIL-125-CS beads is depicted schematically in Scheme 1. First, FeCl_3_ (1.35 g) was mixed with DI water (50 mL) and stirred until evenly dispersed. Then, sodium chitosan (1.5 g) was evenly added to the solution. Finally, MIL-125 (1.5 g) was added into the above solution until well mixed. This mixed solution was added dropwise into a pre-prepared 200 mL solution of 3% Na5P3O10 with constant stirring at room temperature for 2 h. The synthesized MIL-125-CS beads were collected and washed three times with DI water. These beads were then comprehensively characterized using multiple physicochemical techniques to confirm the formation of MIL-125-CS.

### 3.3. Characterization

The surface morphologies of the beads were analyzed using scanning electron microscopy (SEM-S4800, Hitachi, Japan). The functional groups and elemental composition of the adsorbent were monitored by Fourier transform infrared (FTIR) spectroscopy (Tensor II, Bruker, Germany) and X-ray photoelectron spectroscopy (XPS, ESCALAB 250, Shimadzu, Japan). Additionally, thermal gravimetric analysis (TGA) was performed using a thermal gravimetric analyzer (PTC-10A, Rigaku, Lorentz, Japan).

### 3.4. Adsorption Studies

A 0.5-g aliquot of MIL-125-CS was added to 20 mL of a 200-mg/L Pb(II) solution in a 100-mL conical flask. A set of initial Pb(II) concentrations (100–1000 mg/L) was used in the following experiments. In order to study the effect of pH on the adsorption reaction, the pH of the Pb(II) solution was adjusted to 2–7 with HCl (0.1 M) or NaOH (0.1 M). The flask with the adsorbent and Pb(II) solution was shaken at 150 rpm, while maintaining a temperature of 298.2 K during the adsorption process. After the adsorption, the solution was poured into a small brown bottle, and the post-adsorption concentration of Pb(II) was determined.

The removal capacity (*q_e_*_,_ mg/g) of MIL-125-CS was determined by Equation (7):

(7) $$q_{e}=\frac{\left(C_{0}-C_{e}\right)V}{m}$$

where *m* (g) is the weight of MIL-125-CS, *C*_0_ (mg/L) is the initial concentration of Pb(II), *V* (L) is the volume of the Pb(II) solution and *C_e_* (mg/L) is the Pb(II) concentration at equilibrium.

The value of *q_t_* (mg/g) for MIL-125-CS at time *t* was calculated according to Equation (8):

(8) $$q_{t}=\frac{\left(C_{0}-C_{t}\right)V}{m}$$

where *C_t_* (mg/L) indicates the resulting concentration of Pb(II).

The rate (*R*) for the elimination of Pb(II) from the polluted water by MIL-125-CS was calculated using Equation (9):

(9) $$R=\frac{C_{0}-C_{e}}{C_{0}}\times 100\%$$

All experimental results show the average values of three parallel experiments. In order to compare the adsorption performances of MIL-125, chitosan and MIL-125-CS toward Pb(II), aqueous Pb(II) solutions (200 mg/g, 20 mL) were treated with each adsorbent (0.5 g).

## 4. Conclusions

In this study, we hydrothermally prepared MIL-125 following a literature procedure. Then, we mixed it with chitosan and dropped it into a Na_5_P_3_O_10_ solution to form beads under ambient conditions. The synthetic polymer beads were analyzed using SEM, FTIR, XPS and TGA, and a thorough study of the surface characteristics of MIL-125-CS confirmed its successful preparation. Furthermore, the formed beads, as novel adsorbents, were used to adsorb Pb(II) from simulated wastewater. A series of adsorption kinetic studies, adsorption-isotherm modeling and thermodynamic studies led us to conclude that the adsorption process was spontaneous. Notably, 180 min were required to reach equilibrium, eventually leading to a *q*_max_ of the attached Pb(II) of 406.5 mg/g at a pH of 6. After batch experiments and regeneration testing, we finally concluded that the MIL-125-CS beads were an effective and reusable material for the adsorption of Pb(II) from polluted water.

## Figures and Tables

**Table 1:** Kinetic parameters for Pb(II) adsorption on MIL-125-CS at different contact times.

*q_e_*_,*exp*_	Pseudo-First-Order Model			Pseudo-Second-Order Model		
	*q_e_*_,*cal*_	*k*_1_	*R* ^2^	*q_e_*_,*cal*_	*k*_2_	*R* ^2^
99.94	46.45	0.01256	0.97978	102.04	0.0098	0.9999

**Table 2:** The parameters of thermodynamics for Pb(II) removal by the MIL-125-CS beads.

Δ*G* (kJ/mol) *T* (K)			Δ*H* (kJ/mol)	Δ*S* (J/mol∙K)
293.2	298.2	303.2	8.343	28.00
−5.78	−5.92	−6.13		

**Table 3:** Langmuir and Freundlich model parameters for Pb(II) loaded onto MIL-125-CS beads.

*T* (K)	Langmuir Isotherm			Freundlich Isotherm		
	*q_m_* (mg/g)	*b* (L/mg)	*R* ^2^	*K_F_*	*n*	*R* ^2^
298.2	406.50	0.02	0.99077	27.53	1.12	0.94637

**Table 4:** *R_L_* data for Pb(II) adsorption on the MIL-125-CS beads based on the Langmuir model.

*C*_0_ (mg/L)	100	200	300	500	800	1000
*R_L_*	0.33	0.20	0.14	0.090	0.058	0.047
